# Spontaneous nucleation on flat surface by depletion force in colloidal suspension

**DOI:** 10.1038/s41598-021-87626-9

**Published:** 2021-04-26

**Authors:** Nobutomo Nakamura, Yuto Sakamoto, Hirotsugu Ogi

**Affiliations:** 1 Graduate School of Engineering Science, Osaka University, Toyonaka, Osaka 560-8531 Japan; 2 Graduate School of Engineering, Osaka University, Suita, Osaka 565-0871 Japan

**Keywords:** Colloids, Structural properties

## Abstract

Nucleation by sedimentation of colloidal particles on a flat surface is experimentally observed, and effect of attractive depletion force generated by polymers on nucleation is investigated. Sedimentation forms polycrystalline colloidal crystal on a flat surface, and above the threshold polymer concentration, ratio of the spontaneous nucleation increases, resulting in a decrease in the grain size, whereas dependence of the contact angle on the polymer concentration was not observed. We show that the interaction between particles and the flat surface mainly affects the spontaneous nucleation, not the interaction between the particles, and it is demonstrated that the nucleation process can be numerically reproduced using the rate equations.

## Introduction

Since the finding that the phase diagram of a hard-sphere colloidal suspension is similar to that of atomic system^1^, structural dynamic of colloidal suspension has been examined^2–5^, and colloidal system is considered as a promising material for simulating atomic system. In colloidal system, particles are large enough to be observed with a microscope and motion of particles is slow enough to be tracked. In addition, a large system consisting of more than a billion particles is prepared without using the periodic boundary condition. For these features, colloidal system has been attracting attention in the field of material science for modeling atomic system that is experimentally inaccessible.

Among applications of colloidal system, crystallization is a phenomenon that colloidal system has worked effectively^6–12^. In this study, we focus on crystallization especially on a flat surface for modeling growth of a thin film on a substrate. In atomic system, thin film shows several kinds of growth modes depending on material of film and substrate. Volmer–Weber growth, in which deposited atoms diffuse on a substrate, aggregation of atoms causes nucleation, growth of nuclei forms isolated islands, and coalescence of islands forms a polycrystalline continuous film, is a mode often observed in the growth of polycrystalline metallic films^13,14^. The growth process has been monitored using the scanning tunneling microscope^15^, curvature measurement^16,17^, elastic-stiffness measurement^18^, and resistive spectroscopy^19^. However, real-time monitoring of deposited atom is never straightforward, and there are still ambiguities in the dynamics of film growth. Clusters fabricated by deposition is used for gas sensing^20–22^, in which sensitivity depends on the size and shape of clusters, and understanding the formation process of clusters is practically useful.

In colloidal system, one of the methods to form a colloidal crystal is sedimentation of colloidal particles on a flat surface in dilute colloidal suspension. During sedimentation, settled particles diffuse on a surface, and crystalline structure is formed, which is apparently similar to the nucleation process during deposition of atoms. Crystallization on a flat surface has been already investigated using hard-sphere colloidal system^23^, and crystallization during sedimentation has been also discussed by experiments^24–27^ and by computer simulation^28^. During deposition of thin film, film-growth process changes depending on the strength of the interaction between deposited materials and between deposited material and substrate. Therefore, to simulate the film growth using a hard-sphere colloidal system, effect of the attractive interaction on the growth process is investigated and the interaction must be adjusted properly. Then, generation of the attractive interaction by the depletion force is useful. The phase of hard-sphere colloidal system is generally governed by volume fraction of particles. When the depletion force does not exist, spontaneous nucleation does not occur at smaller volume fraction. For example, it is observed that in colloidal systems without attractive interaction crystallization occurs simultaneously in first and second layers, followed by the layer-by-layer crystallization in the third and higher layers^24–26^. Therefore, without giving the attractive interaction, nucleation in the early stage of the deposition cannot be reproduced. In contrast, it is observed that nucleation on substrate occurs in the colloidal system with depletion interaction^27,29^. It is apparently similar to the film growth by deposition. These results indicate that understanding the effect of the depletion interaction on the film-growth process is required for simulating the film growth using the colloidal system.

In this paper, we observe the transition of nucleation behavior from volume-fraction governed nucleation to spontaneous nucleation by changing the attractive force between particles. For generating attractive interaction, attractive depletion force^30^ is employed. We focus on nucleation and following growth, and effect of the attractive depletion force on the nucleation and its growth dynamics is discussed.

## Experiment

Colloidal crystal was grown on a flat surface of a coverslip under gravity. Silica particle with diameter of *d*=1.51 $\upmu$m and polydispersivity of 3% was dispersed in a solution consisting of water, dimethyl sulfoxide, and fluorescein sodium salt. The colloidal suspension was poured in a container whose bottom surface is a flat coverslip, and growth of colloidal crystal was observed from bottom using the confocal laser-scanning microscope. Detail of the container and measurement setup are described elsewhere^11^. Poly(sodium 4-styrenesulfonate) (PSS), molecular weight of 70,000, was added to generate attractive depletion force between particles. After pouring the colloidal suspension into the container, sedimentation was monitored by taking three dimensional stacking images repeatedly. From the stacking images, locations of particles were identified by using the code distributed from Ref.^31^. Then, the results were analyzed using MATLAB.

## Results and discussion

**Figure 1:**
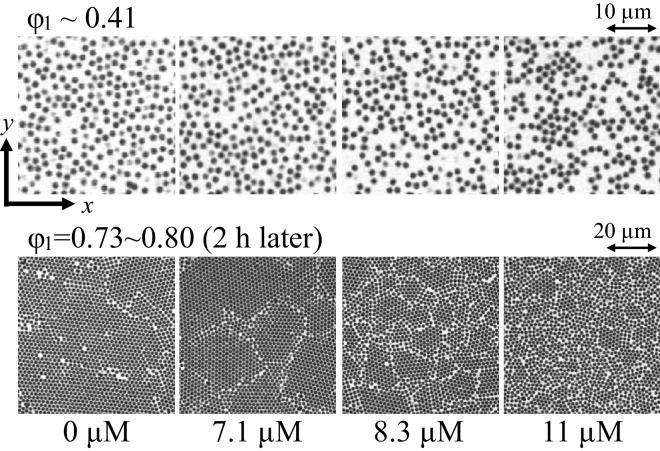
*x*-*y* images of the first layer of sedimented particles on the coverslip.

Figure 1 shows the representative microscope images of the first layer (bottom layer) on the coverslip at the areal density of particle $\phi _{1}$ of $\sim 0.41$ and $0.73\sim 0.80$ (after 2 h sedimentation). We here define the direction perpendicular to the coverslip as the *z*-direction, and the *x* and *y* directions are normal to it. At $\phi _{1}\sim 0.41$, for the polymer concentration $\zeta \le$ 7.1 $\upmu$M particles are dispersed on the coverslip and aggregation is not observed. For $\zeta \ge$ 8.3 $\upmu$M, some particles are in contact with each other, and chain-like structures and aggregates are formed. At $\phi _{1}=0.73 \sim 0.80$, hexagonal packings are observed in all specimens, and their structure is like a polycrystalline metal; there are grains and grain boundaries. For $\zeta \le$ 7.1 $\upmu$M, grain size is apparently independent of $\zeta$, and it becomes smaller as $\zeta$ increases for $\zeta \ge$ 8.3 $\upmu$M. (see also Fig. S1 in supplementary material.)

**Figure 2:**
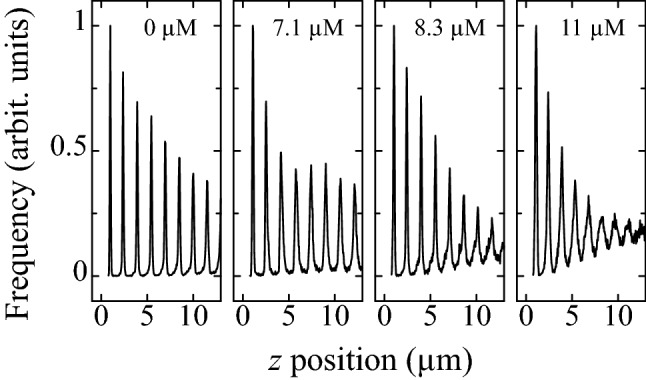
Histogram of the *z* position of the particles after 2-h sedimentation at the PSS concentration of 0.0, 7.1, 8.3, and 11 $\upmu$M.

Figure 2 shows the representative histograms of the *z* position of particles after 2 h sedimentation. In the histograms, peaks appear periodically, which confirms that crystalline structures are formed. For $\zeta \le$ 7.1 $\upmu$M, peaks are observed clearly even at the eighth layer. In contrast, for $\zeta \ge$ 8.3 $\upmu$M, the peak becomes broader as the *z* increases. This result indicates that at higher $\zeta$, disordered structure is formed as the *z* increases. By searching for particles that appear around each peak, we can identify particles that belong to each layer in the following discussion.

We first analyze the structure of the first layer by calculating the two-dimensional order parameter $\psi _{j}^{(6)}$. The order parameter is defined as $\psi _{j}^{(6)}=\frac{1}{N_{j}}\Sigma _{k=1}^{N_{j}}e^{i6\theta _{jk}}$^25^, where $k(=1\ldots N_{j})$ denotes neighbors of particle *j*. $\theta _{jk}$ is the angle between $\mathbf {r}_{jk}=\mathbf {r}_{k}-\mathbf {r}_{j}$ and an arbitrary reference direction in the *x*-*y* plane. Particles with $|\mathbf {r}_{jk}|\le 2.0$ $\upmu$m are identified as neighbors of particle *j*. Absolute value of $\psi ^{(6)}_{j}$ denotes the degree of crystallinity; $|\psi ^{(6)}_{j}|=1$ indicates that particles surrounding a particle form hexagonal packing structure (crystal), and smaller $|\psi ^{(6)}_{j}|$ denotes disordered structure. We here define particles showing $|\psi ^{(6)}_{i}|\ge$0.8 as belonging to closed packs (grains), and those with $|\psi ^{(6)}_{j}|<$0.8 as belonging to disordered regions. The argument of $\psi ^{(6)}_{j}$ indicates the orientation angle of the hexagonal packing formed by the neighbors.

**Figure 3:**
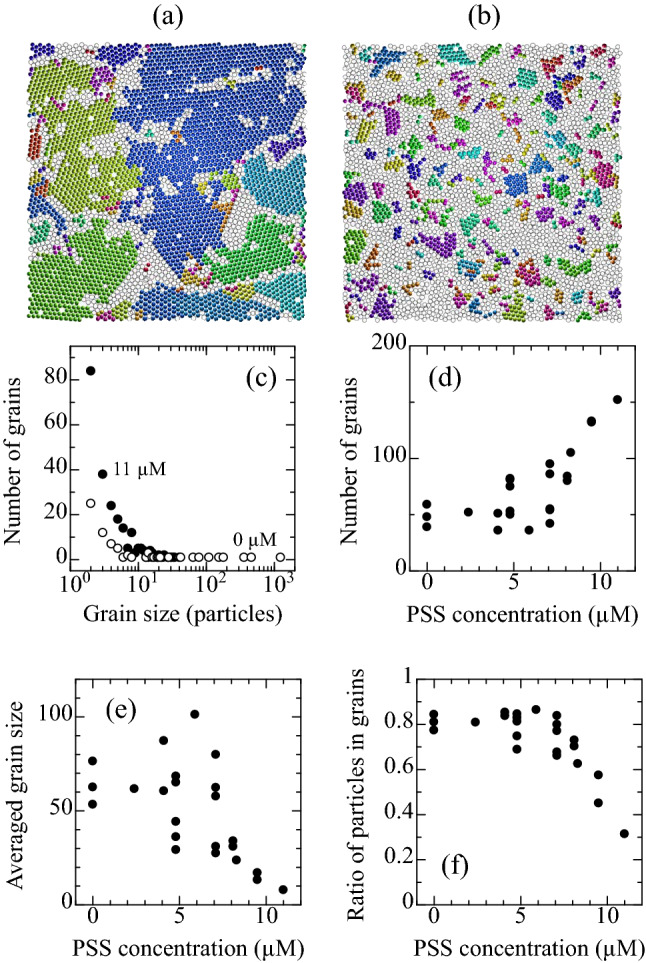
Reconstructed images of the first layer at (**a**) 0.0 and (**b**) 11 $\upmu$M. Each grain is colored with a different color. Particles considered to belong to disordered regions are colored with white. Image size is 98 $\upmu$m $\times$ 98 $\upmu$m. (**c**) Histogram of the grain size at 0.0 (open circle) and 11 $\upmu$M (solid circle). (**d**) The number of grains, (**e**) the averaged grain size, and (**f**) the ratio of particles in grains to the total particles. (**d**)–(**f**) were analyzed for grains consisting of three or more particles in the first layer.

For evaluating the grain size, particles in the first layer with $|\psi ^{(6)}_{j}| \ge 0.8$ are extracted, and their orientation angle is compared to those of neighbors. When difference in the angle is 15 degree or less, the particles were considered to belong to the same grain. Figure 3a,b show grains identified after the sedimentation, and each grain is colored with a different color. Histogram of the size of grains after 2 h sedimentation is plotted in Fig. 3c. The number of grains decreases as the grain size increases. At 0.0 $\upmu$M, a grain larger than 1000 particle was observed. Total number of grains consisting of three or more particles is plotted in Fig. 3d. For $\zeta \le$ 7.1 $\upmu$M, the number of grains is around 50, and it varies widely between experiments. For $\zeta \ge$ 8.1 $\upmu$M, the variation becomes smaller, and the number of grains increases as the $\zeta$ increases. This result confirms that the nucleation rate increases as $\zeta$ increases. In addition, it seems that there is a threshold concentration around $\zeta =$8.0 $\upmu$M. The averaged grain size (the numbers of particles) and ratio of the particles in the grains to the total particles in the first layer were also analyzed as shown in Fig. 3e,f, respectively. In the averaged grain size, the value varies widely for $\zeta \le$ 7.1. However, the variation becomes smaller for $\zeta \ge$ 8.1, and the averaged grain size becomes smaller as $\zeta$ increases. In the ratio of the particles in grains, the threshold concentration is also observed around $\zeta =$8.0 $\upmu$M, above which the ratio decreases as $\zeta$ increases. In the above results, variations of the values below the threshold concentration are generally larger than those above the threshold concentration. It is explained as follows. When the polymer concentration is larger than the threshold concentration, a large number of grains are included in the images taken by the microscope, which enables us to analyze the crystalline structure properly. In contrast, when the polymer concentration is lower than the threshold concentration, grains become larger, and the number of grains analyzed becomes smaller, making the variation in the analyzed data wider.

**Figure 4:**
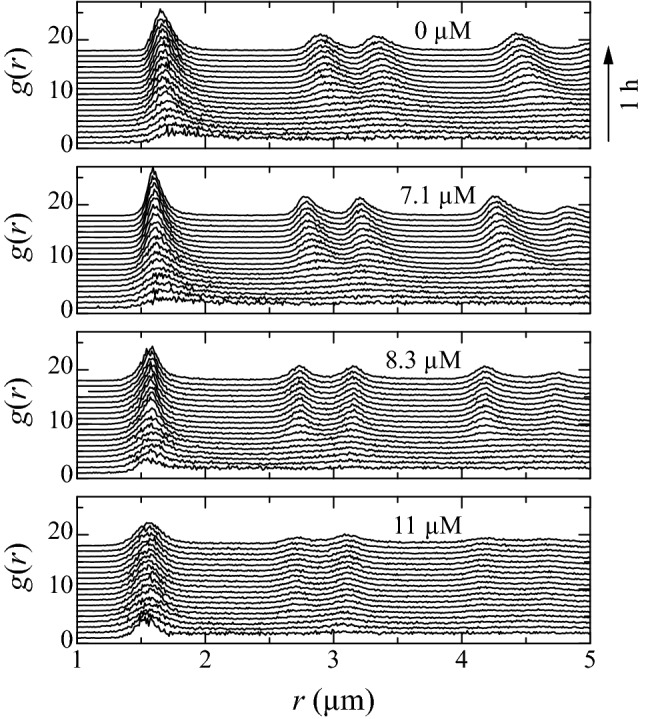
Two-dimensional radial distribution functions in the first layer.

The threshold concentration is observed in the radial distribution functions too. Figure 4 shows two-dimensional radial distribution function in the first layer at different times. For $\zeta \le 7.1$ $\upmu$M, peaks gradually appeared as sedimentation progressed, and inter-particle distance became smaller monotonically. On the other hand, for $\zeta \ge 8.3$ $\upmu$M, a peak from the nearest neighboring particles was observed even at the beginning of the sedimentation, and peak positions were almost unchanged with time.

The above results can be explained by considering that spontaneous nucleation (attractive-force induced nucleation) occurs above the threshold $\zeta$, because the attractive force between particles increases as the polymer concentration increases. However, this interpretation is not enough to explain the result. In supplementary material, motion of the particles on the coverslip obtained with the frame rate of 1 frame/s is shown. The motion was observed with polymer concentration of 0 and 29 $\upmu$M. At 0 $\upmu$M, particles are isolated and move freely on the coverslip. In contrast, at 29 $\upmu$M, particles diffuse over the coverslip to form aggregates. These results can be explained by considering that attractive force between particles is high at 29 $\upmu$M, and particles tend to form aggregates. However, dissociation is also observed on the coverslip, and isolated particles, rather than aggregates, are deposited on the coverslip at 29 $\upmu$M. These results indicate that attractive force between particles is not so strong that dissociation is prevented. On the other hand, interaction between particles and a coverslip changes more clearly depending on the polymer concentration. At $\zeta =0$ $\upmu$M, particles move freely in the *z* direction; some particles disappear from the microscopy images after they arrived at the coverslip surface. However, at $\zeta =29$ $\upmu$M, once the particles arrived at the coverslip surface, it hardly disappeared, indicating that particles are attached to the coverslip strongly. Therefore, it is considered that the threshold $\zeta$ is related to the attractive force between the particle and the coverslip rather than between particles.

The potential between the particles by the depletion force can be calculated from the volume of space in which each polymer cannot move in, and it depends on the diameters of the colloidal particle and polymer and the volume fraction of the polymer^30^. According to this concept, the cohesive energy is expressed as follows $\frac{2\pi k_{B}Tf_{p}}{3}(3aa_{p}^{2}+2a_{p}^{3})$, where $k_{B}$ is the Boltzmann constant, *T* the temperature, *a* and $a_{p}$ the radius of colloidal particles and polymer, respectively, and $f_{p}$ the concentration of polymer. In constant, the cohesive energy between a plate and a particle is calculated as $\frac{\pi k_{B}Tf_{p}}{3}(12aa_{p}^{2}+5a_{p}^{3})$. The hydrodynamics radius of PSS with molecular weight of 70,000 is reported to be in the range of values, 2.86–14.13 nm^32^. This is significantly smaller than the radius of colloidal particles, $a_{p}/a\ll 1$. Then, the cohesive energy between a plate and a particle is approximately twice as large as that between particles. Therefore, the particles start to attach to the coverslip before the particles form aggregates in a solution, and it dominantly affects the crystallization on the coverslip. Validity of this interpretation is confirmed by calculating the radius of PSS from the threshold concentration, $\zeta$ = 8.0 $\upmu$M. Assuming that the adsorption of particles on a coverslip occurs when the cohesive energy becomes larger than the kinetic energy of a particle in the *z* direction, $k_{B}T/2$, hydrodynamic radius of the polymer is calculated to be about 3.3 nm. This value is in the range of the above values, 2.86–14.13 nm, indicating that the above interpretation is reasonable. Thus, adsorption of particles on the coverslip restricts the particles so that they move only on the coverslip, and it increases the frequency of collisions between particles. Therefore, aggregation of particles on the coverslip is accelerated above the threshold concentration.

We also analyze the formation of clusters on substrate using the rate equations referring to the previous work^33^. When deposited atoms arrive on substrate, they diffuse on the surface of substrate and form clusters. The number of clusters with *s* particles per unit area is expressed as follows

1 $$\begin{aligned} \begin{aligned} \frac{dn_{s}}{dt}= \sigma _{s-1} n_{1} n_{s-1} -\sigma _{s} n_{1} n_{s}- \lambda _{s}n_{s} + \lambda _{s+1}n_{s+1} +f \kappa _{s-1} n_{s-1}-f \kappa _{s} n_{s} \end{aligned} \end{aligned}$$

$\sigma _{s}$ denotes the rate at which single particles are attached to the cluster with *s* particles, and $\lambda$ the rate at which single particles detach from a cluster with *s* particles. $\kappa _{s}$ denotes the impingement rate of deposited particles into clusters. *f* denotes the deposition rate. The first and second terms of the right-hand side represent the change of the number of clusters by adsorption of single particles to clusters, and the third and fourth terms represent the change by the desorption. Other terms represent the change by the direct impingement of particles into the clusters. For single particles, its growth rate is expressed as follows,

2 $$\begin{aligned} \frac{dn_{1}}{dt}=f- 2\sigma _{1}n_{1}^{2} - \sum _{s=2}^{\infty }\sigma _{s} n_{1} n_{s}+ 2\lambda _{2}n_{2} + \sum _{s=3}^{\infty } \lambda _{s} n_{s} -\sum _{s=1}^{\infty } f \kappa _{s}. \end{aligned}$$

In the present study, we assume that two-dimensional clusters are formed, and diffusion of clusters with $s>1$ is not considered. $\kappa _{s}$ is the area of clusters ($\kappa _{s}=2\sqrt{3}a^{2}s$, which is the area of the hexagon to which the particle is inscribed). $\sigma _{s}$ and $\lambda _{s}$ are assumed to be proportional to edge length of clusters, $\sigma _{s}= \sigma _{0}\sqrt{s}$ and $\lambda _{s}=\lambda _0\sqrt{s}$, respectively.

**Figure 5:**
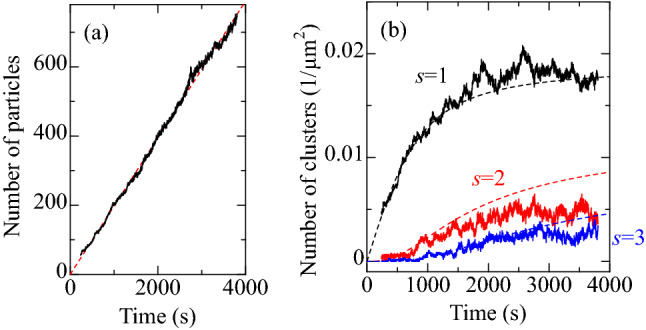
(**a**) Change in the number of particles on the coverslip. Deposition rate was determined from the fitted line (dashed line). (**b**) Changes in the number of clusters with 1, 2, and 3 particles. Solid lines denote the experimental results, and dashed curves denote the calculation results.

Figure 5a shows the change in the number of particles in the first layer with time measured at $\zeta =29$ $\upmu$M. The number of particles increases linearly, and *f* was determined from the slope. In the above equations, $\sigma _{0}$ and $\lambda _{0}$ are fitting parameters. The calculation results at $\sigma _{0}=0.05$ and $\lambda _{0}=0.001$ are shown in Fig. 5b. $n_{1}$ increases monotonically, and it is saturated around 2500 s. Regarding $n_{2}$ and $n_{3}$, it started to increase after a time lag. These behaviors are observed in the experimental results too, and the nucleation on a coverslip is well reproduced using the rate equations.

Finally, we evaluate the shape of grains after 2 h sedimentation at $\zeta$=8.3, 9.5, and 11 $\upmu$M. In this analysis, clusters with (111) orientation in the *z* direction are identified. The two-dimensional order parameter was calculated for particles belonging to each stacking layer referring to the *z*-position histogram (Fig. 2), and particles with $|\psi ^{(6)}_{i}|\ge$0.8 are extracted. After clusters in the bottom layer are identified as shown in Fig. 3a, particles contacting to the clusters are determined among the all of the extracted particles. When the extracted particles have three or more neighboring particles belonging to the same cluster, they are identified as belonging the cluster. This analysis is repeated until no new crystalline particles are found. In Fig. S2 in supplementary material, three-dimensional images of clusters identified using the above analysis are shown.

**Figure 6:**
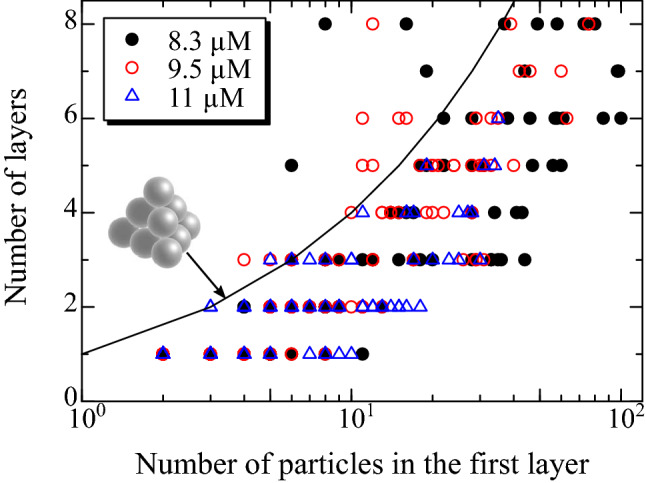
Relationship between the height of clusters and the number of particles in the first layer. The polymer concentration is 8.3 (filled circle), 9.5 (open circle), and 11 $\upmu$M (open triangle). The solid curve denotes the relationship when tetrahedral clusters are formed.

Figure 6 shows the relationship between the number of particles in the first layer of grains and the grains’ height, the number of stacking layers in the *z* direction. As the number of particles in the first layer increases, the height of the grain increases. In hard sphere colloidal crystal, by introducing the depletion force, the interfacial energy increases^27^. It implies that the contact angle of grain would change depending on the polymer concentration $\zeta$. However, notable dependence on $\zeta$ was not observed in Fig. 6. When a tetrahedral of fcc packing is formed, the relationship is expressed as $l=k(k+1)/2$, where *l* is the number of particles in the first layer and *k* the number of stacking layers, which is indicated by the solid curve in Fig. 6. The curve apparently provides the upper limit of the number of layers. Considering that the tetrahedral is formed by staking particles on close-packed layer sequentially, this result indicates that growth of clusters in the *z* direction is caused by deposition of particles on the close-packed layer, and growth by adsorption of particles on triangle surfaces of tetrahedral barely occurs; contact angle of the clusters is independent of the polymer concentration.

## Conclusions

In summary, crystallization by sedimentation on a flat surface was examined, and effect of the attractive depletion force was discussed. A threshold PSS concentration, above which spontaneous nucleation occurs, was observed, and the threshold concentration was related to the depletion force between the particle and coverslip, rather than between particles. Growth of clusters could be reproduced using the rate equations, and it implies that the sedimentation of colloidal particles can be a model system of growth of clusters on substrate by deposition.

## Supplementary Information

Supplementary Information 1.

Supplementary Information 2.

Supplementary Information 3.
