# Supplementary material for: Spontaneous nucleation on flat surface by depletion force in colloidal suspension
Source: Sci Rep. 2021 Apr 26;11:8929. doi: 10.1038/s41598-021-87626-9 (PMC8076313; doi:10.1038/s41598-021-87626-9)
Supplement: Supplementary file 1 — Supplementary Information 1. [file 41598_2021_87626_MOESM1_ESM.pdf]

Supplementary material

Spontaneous nucleation on flat surface by depletion force in colloidal suspension

N. Nakamura, Y. Sakamoto
Graduate School of Engineering Science, Osaka University, Toyonaka, Osaka 560-8531, Japan

H. Ogi
Graduate School of Engineering, Osaka University, Suita, Osaka 565-0871, Japan

S1. Structure of settled particles

In Fig. 1, a part of representative images of settled particles taken with the microscope is shown. Larger images of the specimens are shown in Fig. S1. Image size is 107 μm × 107 μm.

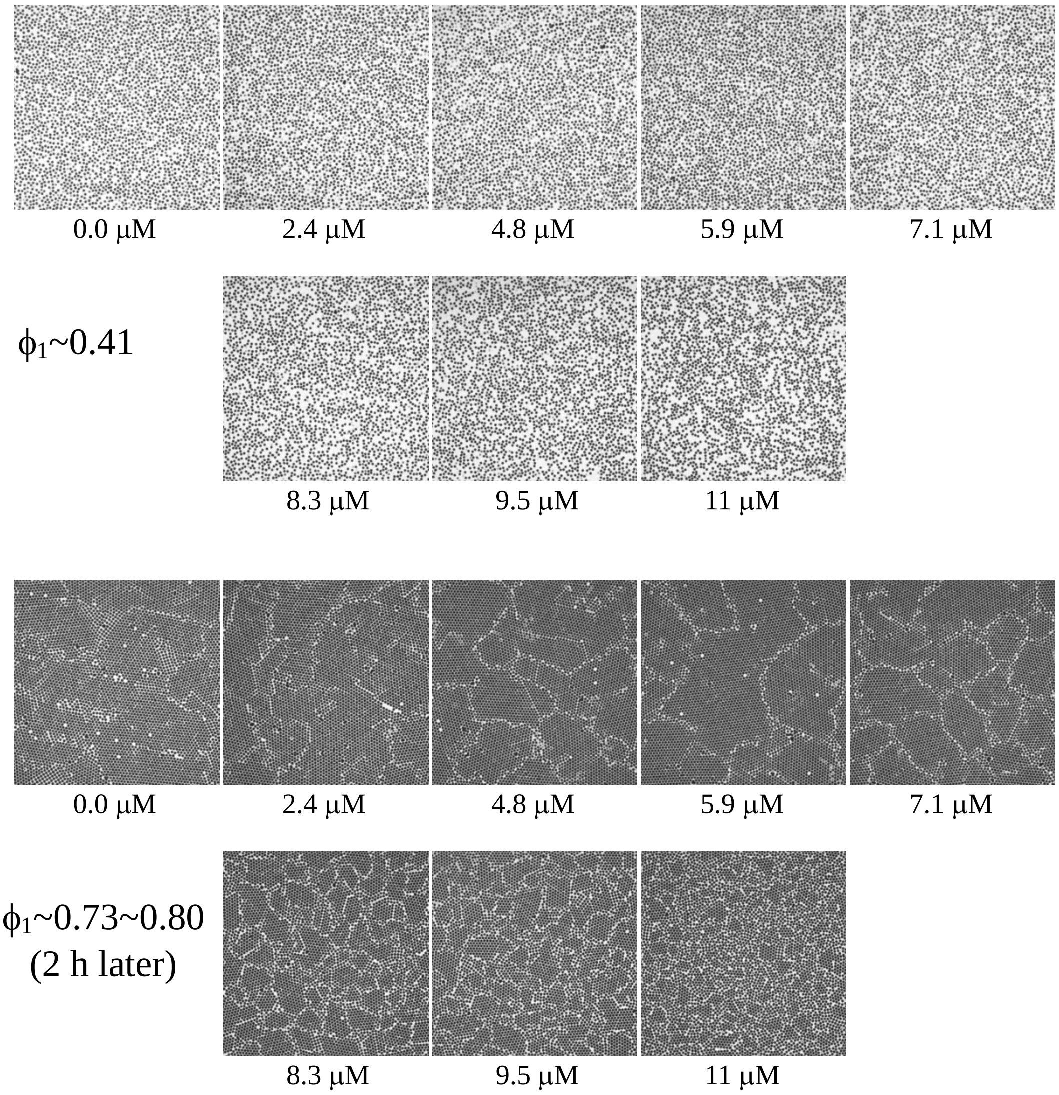

Fig.S1 Images taken with the confocal laser scanning microscope.

S2. Motion of particles on coverslip

To observe the motion of particles on coverslip, 2D images were obtained at 1 frame/s. Movies constructed from the images are shown in Supplementary Information 2 and Supplementary Information 3, in which polymer concentration is 0.0 and 29 μM, respectively. To decrease the file size, one third of all images analyzed are shown, and image quality is also lowered.

S3. 3D structure of clusters

3D structure of clusters on a coverslip is shown in Fig. S2. Image size is 98 μm × 98 μm. When the polymer concentration was 8.3 and 9.5 μM, height of some large clusters was larger than the height of the stacking images taken by the confocal laser-scanning microscopy, and top layers are not plotted in Fig. S2. In Fig. 6, data of larger clusters is not plotted, and this ambiguity is removed.

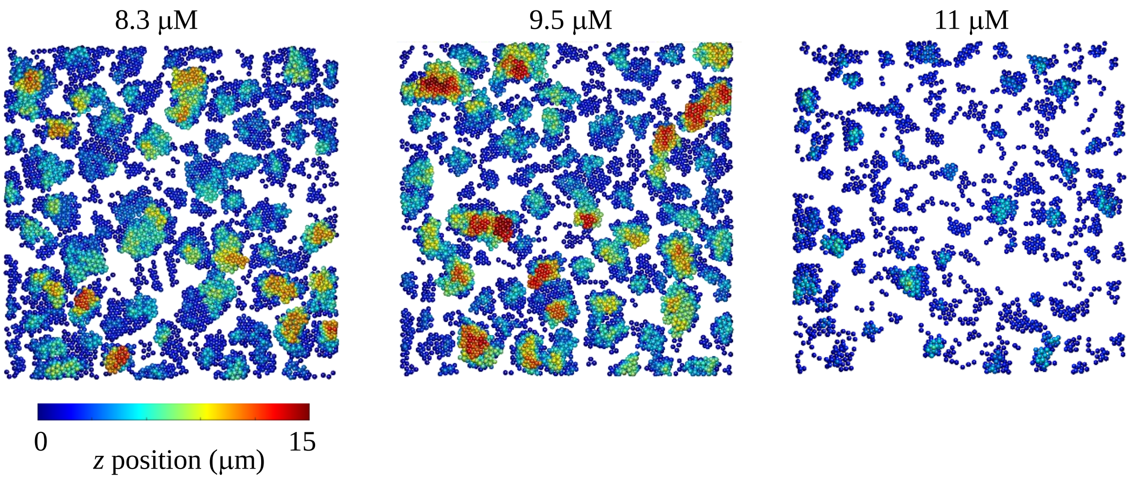

Fig.S2 Reconstructed 3D images of clusters at polymer concentration of 8.3, 9.5, and 11 μM.
